# Factors influencing subjective opinion attribution to conversational robots

**DOI:** 10.3389/frobt.2025.1521169

**Published:** 2025-04-16

**Authors:** Yuki Sakamoto, Takahisa Uchida, Midori Ban, Hiroshi Ishiguro

**Affiliations:** Graduate School of Engineering Science, Osaka University, Osaka, Japan

**Keywords:** subjective opinion, opinion attribution, conversational robot, teleoperation, android, human-robot interaction (HRI)

## Abstract

The usefulness of conversational robots has been demonstrated in various fields. It is suggested that expressing subjective opinions is essential for conversational robots to stimulate users’ willingness to engage in conversation. However, a challenge remains in that users often find it difficult to attribute subjective opinions to robots. Therefore, this study aimed to examine the factors influencing the attribution of subjective opinions to robots. We investigated robot and human factors that may affect subjective opinion attribution to robots. Furthermore, these factors were investigated in four different cases, adopting a combination of the robots’ types and control methods, considering actual scenarios of robot usage. The survey was conducted online, and the participants completed a questionnaire after receiving instructions regarding the robot. The results revealed that the perception of the robots’ sensory capabilities significantly influenced the attribution of subjective opinions in all the surveyed cases. Additionally, in the case of an autonomous small robot, there is a possibility that participants’ self-perception of their judgment abilities might also impact their subjective opinion attribution to the robot. The findings highlight the importance of aligning subjective opinion utterances in conversational robots with user perceptions of the robot’s sensory capabilities. They also emphasized the significance of exploring how users’ self-perceptions influence their perceptions of robots. These insights provide valuable guidance for designing conversational strategies and speech generation in robots that engage in the exchange of subjective opinions with humans.

## 1 Introduction

Robots engaging in conversations with humans have proven to be valuable in various fields, such as education (Kanda et al., 2004) and dementia care for the elderly (Lima et al., 2021). Consequently, conversational robots have become increasingly prevalent in daily life. These conversational systems necessary for such robots can be broadly categorized into two types: task- and non-task-oriented dialogue systems (Chen et al., 2017). Task-oriented dialogue systems are designed to assist users in accomplishing specific tasks, such as providing navigation (Komatani et al., 2003) or making seat reservations (Seneff and Polifroni, 2000). Conversely, non-task-oriented dialogue systems, also known as chatbots, focus on sustaining conversations themselves. For example, studies have aimed to provide human-like and natural responses (Bessho et al., 2012), as well as the development of technologies for detecting dialogue interruptions to sustain interaction (Higashinaka et al., 2016). Moreover, studies on the utilization of large language models to create natural non-task-oriented dialogue systems have increased recently (Zhang et al., 2019; Roller et al., 2020; Thoppilan et al., 2022).

For non-task-oriented dialogue systems to be consistently used by users, it is important not only to enhance the naturalness of interaction but also to stimulate users’ motivation to “want to interact with the robot.” It has been observed that in human-human conversations, when conversation is active, statements of objective facts decrease, whereas subjective utterances expressing personal opinions increase (Tokuhisa and Terashima, 2006). Additionally, the expression of subjective opinions is a type of self-disclosure, which is crucial in fostering intimacy in relationships with others (Altman and Taylor, 1973). Consequently, the importance of endowing non-task-oriented conversational robots with subjective experiences and preferences, as well as the design of mechanisms for utterance generation based on these insights have been emphasized (Minato et al., 2022). These studies have highlighted the importance of conversational robots expressing subjective opinions to increase users’ motivation to interact.

However, people find it challenging to attribute subjective experiences related to value judgments (good or bad) to robots (Sytsma and Machery, 2010), and a similar phenomenon has been observed in the context of subjective opinions in conversation (Uchida et al., 2019, 2024). For example, because typical robots do not consume food, users may struggle to believe statements such as “The cake is delicious” when expressed by a robot. This phenomenon is called “the user does not attribute subjective opinions to the robot (Uchida et al., 2024).” Given that users’ willingness to engage in conversation diminishes when robots express subjective opinions that users cannot attribute to them (Uchida et al., 2019, Uchida et al., 2024), the issue of subjective opinion attribution to robots is worth considering, rather than having robots express subjective opinions similar to humans.

Based on the above, this study aimed to examine the factors influencing the attribution of subjective opinions to conversational robots. By clarifying the factors involved in subjective opinion attribution, this study sought to obtain valuable insights into the design of conversational robots that engage in subjective interactions with humans.

It has been shown that when attributing mental states such as beliefs and intentions to robots, both robot factors, such as behavior, and human factors, such as age, influence this attribution (Thellman et al., 2022). Expressing subjective opinions in conversation can be viewed as an expression of beliefs and intentions; therefore, this study conducted experiments that considered robot and human factors.

Furthermore, considering practical scenarios of robot usage, these factors were examined for four different cases: two types of humanoid robots—an android and a small robot—and two control methods—autonomous and teleoperation. Humanoid robots have frequently been studied in the context of social robots (Erich et al., 2017; Choudhury et al., 2018), and subjective opinion attribution (Uchida et al., 2024). Given the recent prevalence of remotely controlled robots (Takeuchi et al., 2020), we established these four cases for a comprehensive study.

To conduct the examination, participants were recruited through crowdsourcing, and an online survey was conducted. In the online survey, instructions regarding the robots were given to the participants, and they were asked to complete a questionnaire. The questionnaire included questions regarding the attribution of subjective opinions to the presented robots, and questions related to factors influencing opinion attribution. Factor analysis and multiple regression analysis were performed on the survey results to examine the factors influencing the attribution of subjective opinions.

The contributions of this study are as follows. By identifying factors involved in attributing subjective opinions to robots, this work clarifies design considerations crucial for developing conversational robots capable of subjective opinion exchange. An analysis of human and robot factors is presented to identify key aspects impacting the attribution of subjective opinions, which could lead to human-robot interactions considering embodiment. Moreover, conducting experiments across multiple scenarios that reflect realistic robot usage cases provides results that can serve as practical references for deploying conversational robots in real-world settings.

The remainder of this paper is organized as follows. Section 2 discusses previous research related to the attribution of subjective opinions. Section 3 describes the experiments that were conducted. Section 4 presents experimental results. Section 5 presents a discussion based on experimental results, and Section 6 concludes the study.

## 2 Related works

The attribution of subjective opinions, which is the focus of our study, is related to the phenomenon of attributing mental states. The attribution of mental states is a concept introduced to describe the cognitive ability to understand and reflect on the mental states of oneself and others, including beliefs, desires, feelings, and intentions (Brüne et al., 2007). This aids in understanding others during interactions. For example, one strategy that humans use to understand and predict others’ behavior is the intentional stance. This involves assuming that the other party has intentions, making it easier to explain their behavior (Dennett, 1989). Because expressing subjective opinions in conversation can be seen as an expression of beliefs and intentions, the attribution of subjective opinions is related to the attribution of mental states. A review on the attribution of mental states to robots classifies the determinants of mental state attribution into robot factors, such as behavior, and human factors, such as age (Thellman et al., 2022). However, while mental state attribution involves perceiving a robot’s internal states, such as intentions and desires, our study focuses on subjective opinion expressions, which are the robot’s utterances. Therefore, the factors influencing each type of attribution may differ, highlighting the importance of investigating the factors that affect the attribution of subjective opinions.

One study examined the trustworthiness of humanoid robots and mechanical devices as information sources compared with humans (Finkel and Krämer, 2022). In this study, robots and smart speakers were rated lower in terms of trustworthiness and goodwill than humans. Additionally, it was shown that factors, such as anthropomorphism and general reliance on technology, influence the evaluation of a robot’s credibility. This study used indicators related to goodwill as a measure of Source Credibility (McCroskey and Teven, 1999) to assess whether the information provided by robots is broadly trusted. Our study differs in that it specifically focuses on the subjective information expressed by robots.

It may also be possible to relate our study to the expectancy violation theory (EVT). EVT explains individuals’ reactions when their expectations are violated during communication (Burgoon, 2015). In research on human–robot interaction, EVT has been partially supported as applicable (Asavanant and Umemuro, 2021). We can explore the relevance of EVT by examining what expectations users have from robots and how these expectations are violated by the robots’ expression of subjective opinions.

Several studies have focused on attributing subjective opinions to robots. In research investigating the relationship between the attribution of subjective opinions and the willingness to engage in conversation, users’ interests in numerous topics, their degree of attribution of subjective opinion to the robot, and their willingness to engage in conversation were examined (Uchida et al., 2024). It was shown that, not only users’ interest in the topics but also the degree to which opinions were attributed to the robot, influenced their willingness to engage in conversation. While these findings provide valuable insights suggesting that the exchange of subjective opinions between conversational robots and users needs to be carefully designed, the factors involved in the attribution of subjective opinions have not been examined. Additionally, in a study investigating the factors influencing the attribution of subjective opinions to an android robot, it was shown that multiple factors, including the android’s sensing capabilities, affect opinion attribution (Sakamoto et al., 2023). However, a significant limitation of the study is that only an android robot was targeted. This study aims to extend the work by setting up multiple cases that consider different robot usage scenarios.

Additionally, in prior research concerning the acceptability of robots’ subjective utterances, it was found that a robot can enhance the receptivity of its subjective opinions by referring to other robots that hold the same opinion (Mitsuno et al., 2024). Furthermore, it has been demonstrated that when a robot mimics eating food, the credibility of its subjective statements about that food improves (Okafuji et al., 2024). Although these studies have successfully enhanced the acceptability of conversational robots’ utterances, they have not investigated the factors related to opinion attribution.

## 3 Materials and methods

Participants were recruited through crowdsourcing, and an online survey was conducted. In the online survey, participants were first shown an introductory video about a robot, after which they were asked to answer questions regarding the attribution of subjective opinions to the target robots, as well as questions related to the factors that may influence this attribution.

### 3.1 Considered cases

In this study, we considered practical scenarios of robot usage to examine the factors influencing the attribution of subjective opinions in four different cases. For the types of robots, we used the android Geminoid F and the smaller, more mechanically appearing conversational robot, Sota^1^. Additionally, we consider both autonomous control and teleoperation as control methods. Based on these considerations, the following four cases are prepared:

• Autonomous android case
• Autonomous small-robot case
• Teleoperated android case
• Teleoperated small-robot case

This study examined the factors influencing the attribution of subjective opinions within each of the four cases. Consequently, the experiment was designed such that each participant experienced only one case. This approach also helps reduce participant fatigue, which in turn prevents inattentive responses—as such responses increase as surveys progress (Herzog and Bachman, 1981; Bowling et al., 2021)—and maintains the quality of the data collected.

### 3.2 Description of the robot

**FIGURE 1:**
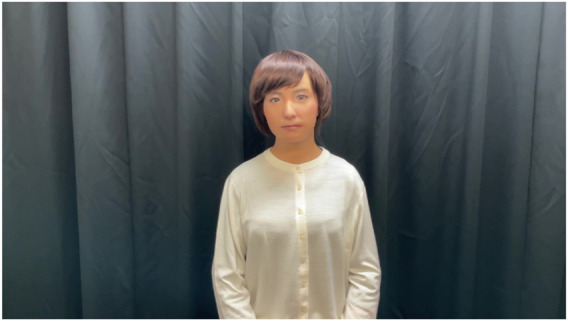
Image from the robot introductory videos shown to participants (autonomous android and teleoperated android cases).

**FIGURE 2:**
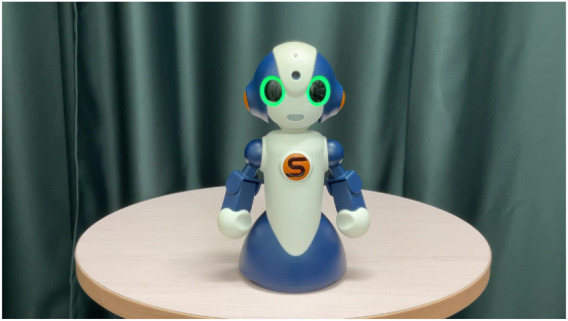
Image from the robot introductory videos shown to participants (autonomous small-robot and teleoperated small-robot cases).

The instructions regarding the robots were provided using video, similar to a related study on the attribution of subjective opinions to robots (Uchida et al., 2024). Before watching the video, the participants received an explanation of the robot. In the autonomous robot cases, an explanatory note stating, “The subject in this photo is a “robot.” It is equipped with AI, allowing it to converse with people face-to-face,” was presented with an image from the corresponding robots’ introductory video. Conversely, in the teleoperated robot cases, the participants were presented with an explanation stating, “The subject in this photo is a “robot.” It is operated remotely by a person, allowing it to converse with people face-to-face,” accompanied by a simple diagram illustrating the remote operation by a human, including an image of the robot. Regarding robot videos, the robots greeted with “Hello. Nice to meet you,” showcasing their speech capabilities. The videos were approximately seven seconds long, with Geminoid F’s speech audio generated using HOYA Corporation’s text-to-speech software^2^ and Sota’s speech audio generated using the voice engine AITalk^3^. Furthermore, involuntary movements of Geminoid F, such as blinking and mouth movements, have been incorporated using existing software (Higashinaka et al., 2021). Sota’s light flashing in the mouth area during the speech was executed using Sota’s built-in function. Figures 1, 2 show the images extracted from the videos.

### 3.3 Methods for data gathering

We used two questionnaires for data gathering. One questionnaire concerned the extent to which participants attribute subjective opinions to robots, while the other addressed factors involved in attributing subjective opinions to robots. In this section, we describe each questionnaire in detail.

#### 3.3.1 Method to measure the attribution of subjective opinions to robots

In the questions regarding the attribution of subjective opinions to the robot, participants were asked, “To what extent do you think this robot can understand the following matters?” They were instructed to assess 90 “matters” on a 5-point scale ranging from “1. Cannot understand at all” to “5. Can understand very well.” In this context, the “matters” being referenced are subjective opinions, which are evaluations made by individuals on a specific target. These opinions are presented as combinations of a topic and an adjective, where the target is the topic. In the experiment, adjectives were provided in nominal form. For example, “the enjoyment of food.” The selected topics and adjectives are explained below.

Twenty-three topics were utilized in the experiment. These topics were selected from those listed in (Yamauchi et al., 2013), which categorized topics in Japanese linguistic activities. During the selection process, the three experimenters chose topics from the life and humanities fields listed in the Practical Japanese Standards, which they judged to appear commonly in everyday conversations. The experimenters were given the following instruction: “For words that you believe commonly appear as topics in everyday conversation, mark them with a 1, and mark those that do not with a 0. Please also mark abstract words that may not appear directly in everyday conversation with a 0. If there are multiple words with similar meanings, give a 1 only to the word you think appears most often in everyday conversation, and assign 0 to the others.” Based on the above instructions, a total of 56 topics were evaluated separately by three experimenters. Twenty-three topics, to which at least two-thirds of the experimenters responded with a 1, were selected. As a result, topics like “general art” were removed. Some expressions were adjusted to ensure a natural tone in conversation during the experiment. The topics explored in the experiments are listed in Table 1.

**TABLE 1:** Topics utilized in the experiment (cited from (Yamauchi et al., 2013)).

Domain	Topics
Life	Food, Alcohol, Clothes, Travel, Sports
	Home, Language, Hometown, Housework
	Home Appliances and Machinery, Party
	Love, Dreams and Goals, Death, Family
	Friendship, Personality, Appearance, School
Humanities	Music, Photography, Movie and Theater
	Fun and Games

Four adjectives were utilized in the experiment. When examining the attribution of subjective opinions to robots, it has been suggested that there is a need to consider the classification of emotional and attributive adjectives in Japanese (Uchida et al., 2019). Using the list of adjectives (Mizukami, 2014) related to topics from Yamauchi et al. (2013), and the classifications of emotional and attributive adjectives (Nishio, 1972; Utsumi et al., 1993), the adjectives “interesting” and “fun” were used as emotional adjectives, while “good” and “beautiful” were used as attributive adjectives.

Among the combinations of topics and adjectives, questions about “the goodness of death” and “the interestingness of death”were excluded as more than half of the participants in prior research (Sakamoto et al., 2023) indicated these did not make sense in Japanese. Consequently, participants were presented with questions regarding 90 “matters.”

#### 3.3.2 Method to investigate factors influencing attribution of subjective opinions to robots

The attribution of mental states to robots is influenced by both robot factors, such as behavior, and human factors, such as age, influence attribution (Thellman et al., 2022). Similarly, prior studies on the attribution of subjective opinions to an android robot have suggested that both robot and human factors impact attribution (Sakamoto et al., 2023). The study by Sakamoto et al. clarified that factors such as the attribution of subjective opinions to participants themselves and perception of the robot’s sensory capabilities, affect subjective opinion attribution to an android robot. In the current study, the questionnaire categories explored as factors influencing the attribution of subjective opinions to robots were determined based on this previous research (Sakamoto et al., 2023). Below, we explain specific questions for each category. The question categories surveyed in this study are shown in Table 2.

**TABLE 2:** Question categories and their classifications examined as factors involved in the attribution of subjective opinions to robots used in experiments.

Factor	Question category
Robot factors	Sensory capabilities of the robot
	Whether the robot is perceived as a tool or a companion
	Open-ended impression evaluation of the robot
Human factors	Attribution of subjective opinions to participants themselves
	Knowledge of the robot
	Participants’ thought processes
	Participants’ age
	Participants’ gender

##### 3.3.2.1 Sensory capabilities of the robot

Given that sensors are necessary for robots to perceive objects, it is reasonable to assume that many people believe that sensors are necessary for robots to have opinions. Therefore, five questions were prepared regarding the robot’s sensory capabilities. Participants were asked, “To what extent do you think this robot possesses the following senses?” and evaluated each of the senses—vision, hearing, touch, taste, and smell—on a 5-point scale ranging from “not at all” to “very much.” In this question, Option 1 is interpreted as the robot having no such sense, meaning it lacks the sensor, while Option 5 is interpreted as the robot having sensory abilities comparable to humans. Given that a robot’s sensory capabilities are still developing, using a 5-point scale, including intermediate values, allows for a better understanding of participants’ perceptions of the robot’s sensory capabilities. In this study, labels were assigned only to the lowest and highest response options, while the intermediate options were left unlabeled. This approach is used in questionnaires that measure sensory intensity (e.g., a questionnaire assessing the vividness of mental imagery related to the five senses (Andrade et al., 2014)).

##### 3.3.2.2 Whether the robot is perceived as a tool or companion

Generally, individuals tend to empathize more easily with robots that resemble humans (Riek et al., 2009), and empathy may influence how mental states are attributed (Gena et al., 2023). Therefore, to assess whether participants perceive the robot as human-like, they responded using labels, such as “tool” and “companion,” which are commonly utilized to describe attitudes toward robots (Bryson, 2010; Danaher, 2019). Then, a single question was asked: “To what extent do you perceive this robot as a ‘tool’ or a ‘companion’?” and rated their responses on a 5-point scale. This questionnaire is related to how people perceive the identity of robots and is classified as a robot factor according to the categorization in previous research (Thellman et al., 2022).

##### 3.3.2.3 Open-ended impression evaluation of the robot

To assess the impressions participants formed of the presented robot, after showing the introductory video of the robot, participants were asked, ‘What did you think after watching this video? Please describe your impressions freely.’ Participants were then asked to provide open-ended responses.

##### 3.3.2.4 Attribution of subjective opinions to participants themselves

People tend to infer others’ knowledge based on their own knowledge (Nickerson, 1999), and it has been suggested that they may reference their own or others’ knowledge when considering non-human agents (Epley et al., 2007). To account for participants’ self-perceptions, questions were included regarding the attribution of subjective opinions to themselves. These questions were similar to the one on subjective opinion attribution to robots, with “robot” replaced by “you” in the question text, resulting in a total of 90 questions.

##### 3.3.2.5 Knowledge of the robot

To address the potential influence of prior knowledge of the target robot on opinion attribution, a single question was posed, asking participants to assess their familiarity with the robot on a 3-point scale ranging from “1. I have never seen it before” to “3. I have seen and communicated with it in person.” This questionnaire can be considered a type of interaction history and is classified as a human factor according to the categorization in previous research (Thellman et al., 2022).

##### 3.3.2.6 Participants’ thought process

The widely recognized dual-process theory suggests that human thought can be fast and intuitive or slow and deliberative (Evans and Stanovich, 2013). In previous research grounded in dual process theory, which examined the phenomenon of people perceiving a mind in agents such as robots that do not possess a mind, it was demonstrated that the factors mediating mind perception vary depending on which of the two thought processes is activated (Koban and Wieringa, 2024). Therefore, the type of thought process of participants may influence the factors involved in attributing subjective opinions to robots. To investigate this possibility, questions regarding the thought process were prepared. Using the labels “Rational” and “Intuitive,” which are commonly used (Witteman et al., 2009; Hamilton et al., 2016), a single question was posed: “As words describing you, which do you think applies more: ‘rational’ or ‘intuitive’?” The participants rated their responses on a five-point scale.

##### 3.3.2.7 Participants’ age and gender

Additionally, questions regarding age and gender were included as basic attributes of the participants. Participants were asked, “Please enter your age using half-width numbers,” and “Please indicate your gender.”

Moreover, owing to the numerous questions, a directed questions scale (DQS) (Maniaci and Rogge, 2014) was incorporated to detect satisficing (Krosnick, 1991).

### 3.4 Procedure

The participants were recruited through crowdsourcing. CrowdWorks^4^ was the platform used for crowdsourcing. Participants were randomly assigned to one of four cases. After receiving instructions regarding the target robot, which included one video, in their assigned case, they completed a questionnaire. Additionally, in all cases, a question was posed after viewing the video to validate whether participants listened to the robot’s speech. This involved participants writing the content of the robot’s speech. The participants were observers who did not interact with the robot or with each other. The online survey took approximately 30 min to complete, and participants were compensated with an honorarium of JPY 550. All participants provided informed consent prior to the commencement of the study, which was approved by the Ethics Committee of Osaka University, Japan.

## 4 Results

Participants who responded to more than two cases, answered the DQS inserted in the questionnaire incorrectly, or provided incorrect answers to questions regarding the audio in the robot introductory video were excluded from the analysis. Based on these criteria, the participants included in the analysis were as follows: autonomous android case: 98 participants (average age =40.12,SD=9.44 , 53 males, 44 females, 1 unanswered); autonomous small-robot case: 98 participants (average age =39.26,SD=8.20 , 53 males, 45 females); teleoperated android case: 96 participants (average age =40.40,SD=9.57 , 56 males, 39 females, 1 unanswered); teleoperated small-robot case: 97 participants (average age =39.56,SD=8.47 , 52 males, 45 females).

This section presents the results of the analysis. After reviewing the internal consistency between the questions, a multiple regression analysis was conducted to examine the factors influencing the attribution of subjective opinions to the robot. Responses to the questionnaire items related to the attribution of subjective opinions to the robot were utilized as dependent variables, whereas responses to the other questionnaire items (excluding the open-ended impression evaluation of the robot) served as independent variables.

First, Cronbach’s alpha coefficients were calculated for each adjective in questions related to the attribution of subjective opinions. Owing to the confirmation of sufficiently high internal consistency (α=.907−.975) , averages were computed for each adjective, and these values were used as variables.

Subsequently, based on the factors from prior research (Sakamoto et al., 2023), factor analyses using the maximum likelihood method were conducted for both independent and dependent variables. For the independent variables, the initial eigenvalues were 3.935, 2.644, 1.535, and 1.021 for the first through the fourth factors, respectively. Because the initial eigenvalues for up to the fourth factor were above 1.0, factor analysis was performed by reducing the number of factors from four, using maximum likelihood and Promax rotation. This resulted in three factors for the independent variables with eigenvalues greater than 1 and factor loadings greater than 0.5, leading to the conclusion that a three-factor structure was appropriate. The total variance explained by these three factors was 85.8%. For the dependent variables, the initial eigenvalue for the first factor was 3.780, and the proportion of variance explained was 94.5%. The initial eigenvalue for the first factor was greater than 1.0. Factor structures are presented in Tables 3, 4.

**TABLE 3:** Factor structure of the independent variables.

Variables		1st	2nd	3rd
Subjective opinion attribution to participants	Interesting	0.961	0.005	−0.017
	Enjoyable	0.948	0.001	−0.004
	Good	0.946	−0.071	0.028
	Beautiful	0.919	0.069	−0.004
Perception of robot sensor capability	Smell	0.000	0.926	−0.064
	Taste	−0.008	0.914	−0.068
	Touch	0.025	0.581	0.241
	Hearing	−0.039	0.019	0.956
	Vision	0.043	−0.002	0.712

**TABLE 4:** Factor structure of the dependent variables.

Variables		1st
Subjective opinion attribution to robot	Good	0.982
	Interesting	0.970
	Enjoyable	0.956
	Beautiful	0.944

The first factor of the independent variables, labeled “Cognition of Participants’ Judgment Ability,” comprised items related to participants attributing subjective opinions to themselves, reflecting their belief in judgment abilities. The second factor of the independent variables comprised items related to the sensory capabilities of the robots. In interactive systems, those that utilize visual and auditory modalities are prevalent; however, systems that engage taste, smell, and touch are in the developmental stages (Obrist et al., 2016). Therefore, the second factor, encompassing smell, taste, and touch senses, was labeled “Cognition of Senses Difficult for Robots to Possess.” Conversely, the third factor, which comprises hearing and vision senses, has been designated as “Cognition of Senses Easy for Robots to Possess.”

Next, multiple regression analysis using a forced entry method was conducted to examine the relationship between the dependent and independent variables based on the factor scores for each of the four cases. Additionally, when calculating the variance inflation factor (VIF), it was found that the VIF was less than two for all models, indicating that multicollinearity was not an issue. The Shapiro-Wilk test was also performed, and normality was confirmed ( p>.05 for all models). Furthermore, the Breusch-Pagan test indicated that homoscedasticity was not present in the cases of the autonomous small robot and the teleoperated android (p<.05) . Therefore, for these two cases, log transformation was applied to the dependent variables before performing multiple regression analysis.

**FIGURE 3:**
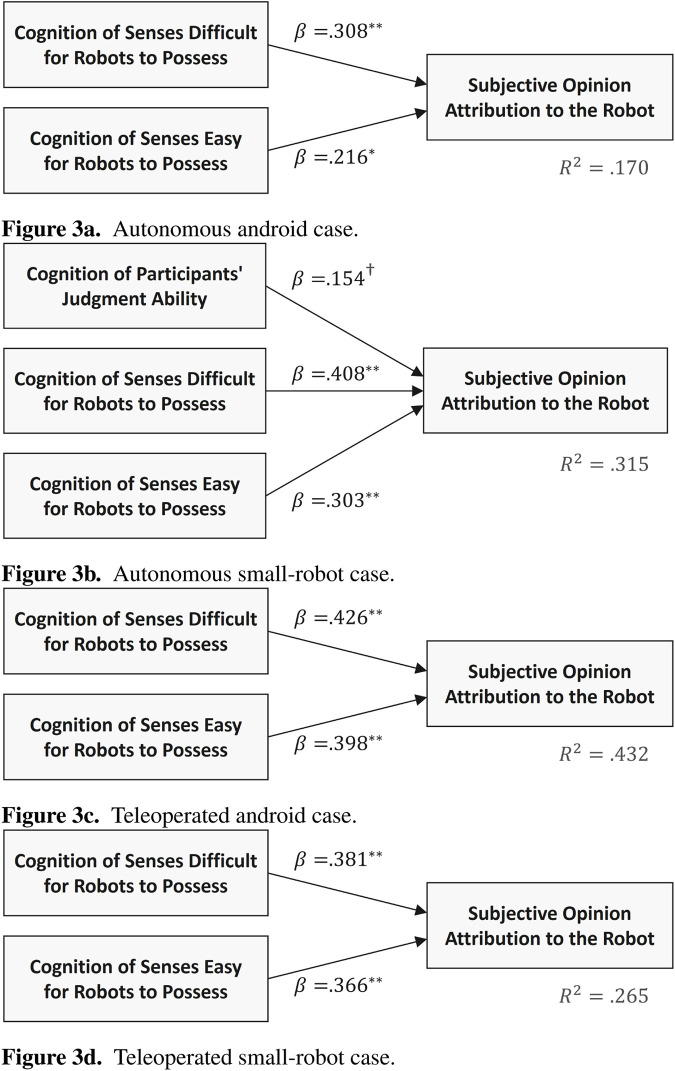
Path diagrams of multiple regression analysis for each case (†p<.1,∗p<.05,∗∗p<.01) . **(a)** Autonomous android case. **(b)** Autonomous small-robot case. **(c)** Teleoperated android case. **(d)** Teleoperated small-robot case.

Multiple regression analysis results indicated that the model was significant in all cases ( p<.001 for all models). The paths along which the effects were observed for each case are shown in Figure 3. In the autonomous android case, significant paths were observed for the second independent factor (Cognition of Senses Difficult for Robots to Possess) on the dependent variable (β=.308,p<.01) and the third independent factor (Cognition of Senses Easy for Robots to Possess) (β=.216,p<.05) . In the teleoperated android case, both the second factor of the independent variables (β=.426,p<.01) and the third factor of the independent variables (β=.398,p<.01) had a significant effect on the dependent variable. In the teleoperated small-robot case, both the second (β=.381,p<.01) and third (β=.366,p<.01) independent factors demonstrated significance with respect to the dependent variable. On the other hand, in the autonomous small-robot case, the regression coefficient for the first factor of the independent variables was β=.154(p<.1) , the second factor was β=.408(p<.01) , and the third factor was β=.303(p<.01) . This indicates a marginally significant for the influence of the first factor and significant effects for the second and third factors.

**FIGURE 4:**
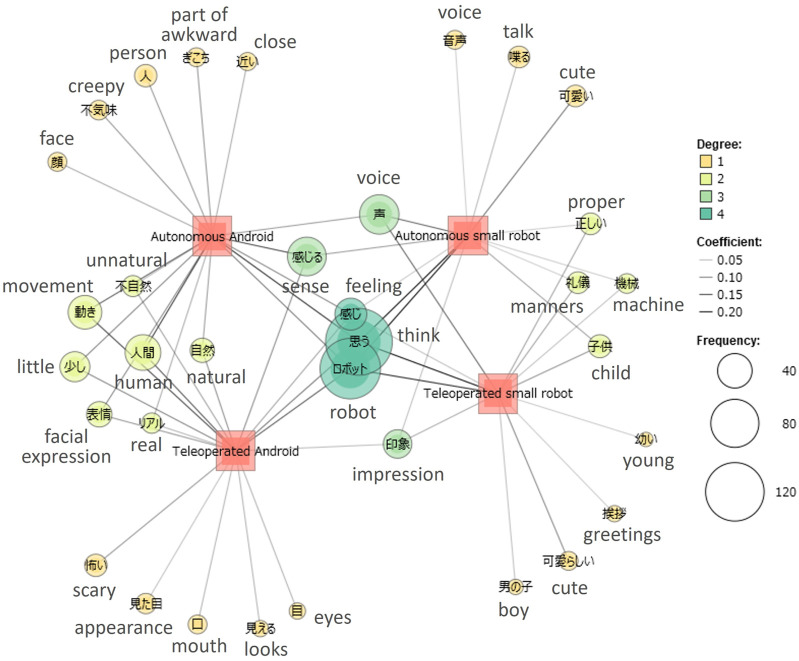
Co-occurrence network analysis of open-ended responses with cases as external variables (degree represents the number of external variables with which the extracted word co-occurs; coefficient represents the level of co-occurrence expressed by the Jaccard coefficient; and frequency indicates the occurrence frequency of the extracted words). English translations are added to each word.

Text mining was performed on the open-ended impression evaluation items for the robots using KH Coder^5^. KH Coder is text mining software that supports Japanese and can appropriately segment sentences into words and calculate word frequency. To reveal the relationship between the frequency of extracted words and external variables (four cases), a co-occurrence network analysis was conducted between the extracted words and external variables. The results are shown in Figure 4.

Furthermore, considering that the human-likeness of robots influences the attribution of mental states (Krach et al., 2008; Martini et al., 2016; Manzi et al., 2020), an analysis was conducted on the ratio of impressions of “human” versus “machine” for the open-ended impression evaluation items regarding the robots. The analysis focused on the frequency of words related to “human” and “machine” in the free-text responses.

To conduct the analysis, words were extracted from open-ended comments using KH Coder. The extracted words were then vectorized using OpenAI’s text-embedding-3-large model^6^. By comparing the vectors of the extracted words with those of “human” and “machine” using cosine similarity, words that surpassed a predetermined threshold (set at 0.5) were classified as either “human-related” or “machine-related” words. Following the classification, the frequencies of human-” and machine-related” words were tallied separately, and their ratio were computed. The results are listed in Table 5.

**TABLE 5:** Frequency and ratio of “Human” and “Machine”-related words in open-ended evaluation items.

	Autonomous Android	Autonomous Small robot	Teleoperated Android	Teleoperated Small robot
Machine-related word count (A)	28	45	31	41
Human-related word count (B)	32	4	21	6
Ratio (A/B)	0.875	11.25	1.48	6.83

In the autonomous android case, the frequency of “human-related” words was 32, surpassing the 28 “machine-related” words. Conversely, in the autonomous small-robot case, “machine-related” words were more prevalent with 45, compared with four occurrences of “human-related” words. In the teleoperated android case, “machine-related” words appeared 31 times, and 21 occurrences of “human-related” words. In the teleoperated small-robot case, “machine-related” words were more frequent with 41 instances, whereas “human-related” words appeared six times. The ratio of “machine” to “human” words in each case varied, with values of 0.875 for the autonomous android case, 11.25 for the autonomous small-robot case, 1.48 for the teleoperated android case, and 6.83 for the teleoperated small-robot case.

## 5 Discussion

This study aimed to examine the factors influencing the attribution of subjective opinions to robots under four cases related to the robot’s types and method of operation. The results showed that, across all cases, the user perception of the robot’s sensory capabilities significantly impacted the attribution of subjective opinions. This suggests that enhancing users’ awareness of the robot’s sensory systems is crucial in addressing issues of attribution of subjective opinions. This finding aligns with previous research suggesting that a robot’s subjective statements become more credible by simulating eating behavior to create a shared pseudo-dining experience with users (Okafuji et al., 2024). In this study, the confirmation that user perception of a robot’s senses significantly affects subjective opinion attribution suggests that users’ perception of the robot’s sensory experiences and actions may impact the attribution of subjective opinions. While the acceptability of robot speech is often discussed with its “human-likeness” (Schreibelmayr and Mara, 2022), the results of this study indicate the importance of not only advanced cognitive behaviors and emotional expressions but also the users’ perceptions of the robot’s sensory capabilities.

Additionally, in the autonomous small-robot case, there was a marginally significant indication that participants’ perceptions of their own judgment abilities also influenced the attribution of subjective opinions. In the open-ended impression evaluation items, results from the co-occurrence network analysis of text data (Figure 4) reveal that in the small robot cases, words such as “machine” and “child” co-occur, whereas in the android cases, words such as “human” and “facial expression” co-occur. This suggests that participants may have perceived the small robot as more mechanical and childlike than the android robot. Furthermore, as another result highlighting the distinctiveness of the autonomous small robot, as shown in Table 5, the words related to “machine” appeared most frequently in the autonomous small-robot case compared to other cases, indicating that participants might have especially recognized the autonomous small robot as a mechanical entity. These results shed light on how perceiving a robot as mechanical can impact the attribution of subjective opinions. While previous research has demonstrated that imbuing robots with human-like characteristics can enhance interactions (Schreibelmayr and Mara, 2022), the impact of robots perceived as “mechanical” on user cognition has not been extensively explored. Previous research suggests that when considering non-human agents, human or self-related knowledge may be referenced because humans lack immediate access to the phenomenological experiences of non-human agents owing to physical constraints (Epley et al., 2007). As the autonomous small robot was perceived as mechanical, the participants may have inferred the robot’s judgment abilities based on their own, potentially influencing the attribution of subjective opinions to the robot. This study’s findings underscore the importance of further exploring how robots, perceived as “mechanical entities,” are received and understood.

Moreover, the factor structure related to sensory capabilities, as shown in Table 3, may evolve with technological advancements. In this study, hearing and vision were categorized under “Cognition of Senses Easy for Robots to Possess,” whereas smell, taste, and touch were categorized under “Cognition of Senses Difficult for Robots to Possess.” However, robots equipped with tactile sensors are in development for touch interactions (Silvera-Tawil et al., 2015; Lin et al., 2021; Burns et al., 2022), and research on endowing robots with taste (Sochacki et al., 2021) and smell (Marques et al., 2002) is underway. These technological advancements are likely to alter user perceptions of robot sensory capabilities, necessitating ongoing evaluation.

This study has some limitations. The experiment was conducted through an online survey, depriving participants of the opportunity to interact face-to-face with robots, which could have influenced the results. Instead of interacting with the robot, participants watched the videos of the robot online. An observer’s perspective was gathered in this study. In the future, studies engaging humans and robots in conversations exchanging subjective opinions (virtually or in person) need to be conducted. This will allow the evaluation of the resulted factors from this study before integrating these into a robot intended for human-robot interaction (HRI). In actual usage scenarios, the factors influencing the attribution of subjective opinions to robots can change as interactions progress. Previous research has compared direct interactions with robots-to-video interactions, demonstrating a high level of consistency between the outcomes (Woods et al., 2006). However, another study suggests that physical and video-mediated interactions might result in differences in human trust of robots (Bainbridge et al., 2008). Therefore, future research should include validation in scenarios involving face-to-face interactions with robots to more accurately simulate real-life conversations.

Furthermore, regarding the instruction method, participants watched the video only before responding to the questionnaire regarding the attribution of subjective opinions for the 90 matters. As described in the Procedure section, the total duration of the experiment was approximately 30 min, with roughly half of the questionnaire items relating to the robot. Thus, it can be estimated that participants spent about 15 min responding to questions concerning subjective opinion attribution about the robot; we assume that participants could recall the scenario adequately during this duration. Additionally, we deliberately showed the robot video only once at the start, because presenting the video again during the questionnaire might have provided participants with an impression different from their initial viewing. However, future studies should also examine how the timing of stimulus presentation influences experimental outcomes.

In this experiment, adjectives were selected based on previous studies (Mizukami, 2014; Nishio, 1972; Utsumi et al., 1993), and were limited to positive ones. It is also important to examine how the factors influencing the attribution of subjective opinions to robots change when more negative or neutral adjectives are used, which remains a challenge for future research.

In this study, a direct comparison between the four cases was not conducted. This is because the objective of the study was to investigate the factors influencing the attribution of subjective opinions in each of the four different cases, which were considered in the context of actual scenarios of robot use. Furthermore, when conducting analysis, some models were log-transformed following homoscedasticity tests, which resulted in different interpretations of coefficients across models, making discussions of coefficients between models difficult. However, a direct comparison between cases could be useful for clarifying the differences in the factors influencing subjective opinion attribution across different cases. As a future prospect, experiments aimed at comparing cases should be conducted to further develop the findings of this study.

Furthermore, the field of human–robot interaction has highlighted the impact of cultural differences. For example, Japanese individuals tend to exhibit a more positive attitude toward human-like robots (humanoid robots) than other nationals (Haring et al., 2014; Nomura et al., 2015). Conversely, some findings indicate that Japanese attitudes toward robots are not necessarily more positive than those of other nationals (Bartneck et al., 2007; MacDorman et al., 2009). In studies related to the attribution of mental states, it has been reported that Japanese people, compared to Australians, tend to perceive robots with higher animacy, and evaluate them as more intelligent and safer (Haring et al., 2015). Reports also suggest that the mind structures attributed by young Japanese people to non-living entities are similar to those in other cultures (Ishii and Watanabe, 2019). Thus, while there is no established perspective on the impact of cultural differences on human–robot interaction, it may be necessary to consider cultural differences in the attribution of subjective opinions. This experiment focused on humanoid robots, and was conducted in Japan. Similar experiments should be conducted in other languages and countries to explore potential differences in results owing to cultural influences.

## 6 Conclusion

This study explored the factors influencing the attribution of subjective opinions to conversational robots. Both robot and human factors were examined as contributors to subjective opinion attribution to robots. Furthermore, to simulate real-world usage scenarios, these factors were examined in four different cases, considering the robot’s type (android robot and small robot) and control method (autonomous and teleoperated). The results of the online survey indicated that across all cases, the perception of the robot’s sensory capabilities influenced the attribution of subjective opinions to the robots. Additionally, in the case of an autonomous small robot, there was a marginally significant indication suggesting that participants’ self-perception—particularly their cognition of their own judgment ability—might influence their attribution of subjective opinions to the robot. These findings offer valuable insights for developing dialogue strategies and speech generation in conversational robots that exchange subjective opinions with humans.

## Data Availability

The raw data supporting the conclusions of this article will be made available by the authors, without undue reservation.

## Footnotes

1
https://www.vstone.co.jp/products/sota/

2
https://readspeaker.jp/

3
https://www.ai-j.jp/

4
https://crowdworks.jp/

5
https://www.screen.co.jp/as/solution/khcoder

6
https://openai.com/index/new-embedding-models-and-api-updates/
